# Exhausted T cells hijacking the cancer-immunity cycle: Assets and liabilities

**DOI:** 10.3389/fimmu.2023.1151632

**Published:** 2023-04-14

**Authors:** Anna E. Brunell, Riitta Lahesmaa, Anu Autio, Anil K. Thotakura

**Affiliations:** ^1^ Turku Bioscience Centre, University of Turku and Åbo Akademi University, Turku, Finland; ^2^ Immuno-Oncology, Oncology Research, Orion Corporation, Turku, Finland

**Keywords:** T cell exhaustion, cancer-immunity cycle, immunotherapy, functional adaption, T cell dysfunction

## Abstract

T cell exhaustion is an alternative differentiation path of T cells, sometimes described as a dysfunction. During the last decade, insights of T cell exhaustion acting as a bottle neck in the field of cancer immunotherapy have undoubtedly provoked attention. One of the main drivers of T cell exhaustion is prolonged antigen presentation, a prerequisite in the cancer-immunity cycle. The umbrella term “T cell exhaustion” comprises various stages of T cell functionalities, describing the dynamic, one-way exhaustion process. Together these qualities of T cells at the exhaustion continuum can enable tumor clearance, but if the exhaustion acquired timeframe is exceeded, tumor cells have increased possibilities of escaping immune system surveillance. This could be considered a tipping point where exhausted T cells switch from an asset to a liability. In this review, the contrary role of exhausted T cells is discussed.

## Graphical Abstract

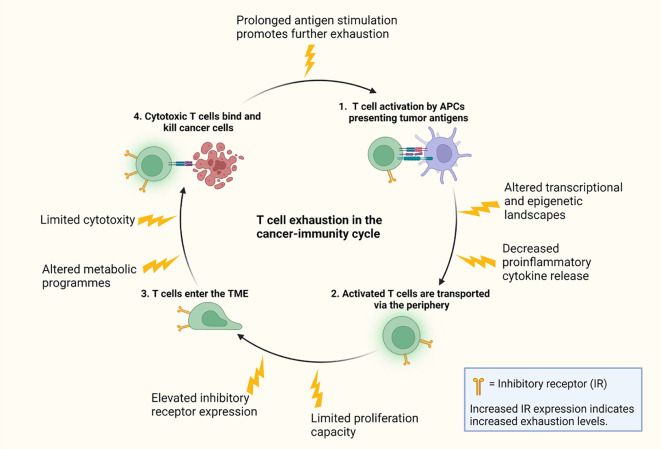

## 1 Introduction

### 1.1 T cell exhaustion

T cell exhaustion can be defined as a physiological state of T cells displaying phenotypical and functional changes, such as eventual loss of proliferation capacity and effector functions, changes in cell metabolism and transcription, decreased production of cytokines and elevated expression of inhibitory receptors (IRs) (1–3). Exhausted T cells are epigenetically altered (4), having approximately 6000 different accessible chromatin regions compared to effector and memory T cells, indicating that exhausted T cells may be considered a distinct cell type (5, 6). The main drivers of T cell exhaustion such as prolonged T cell receptor (TCR) stimulation by antigens, exposure to suppressive cytokines and tumor mediated immunosuppressive metabolic byproducts, were previously thought to be located mainly in the tumor microenvironment (TME) (7). More recent studies provide evidence that the exhaustion journey of T cells is initiated already in the lymph nodes (LNs) through interaction between antigen presenting cell (APC)-presented tumor antigens and T cells (8).

**Figure 1:**
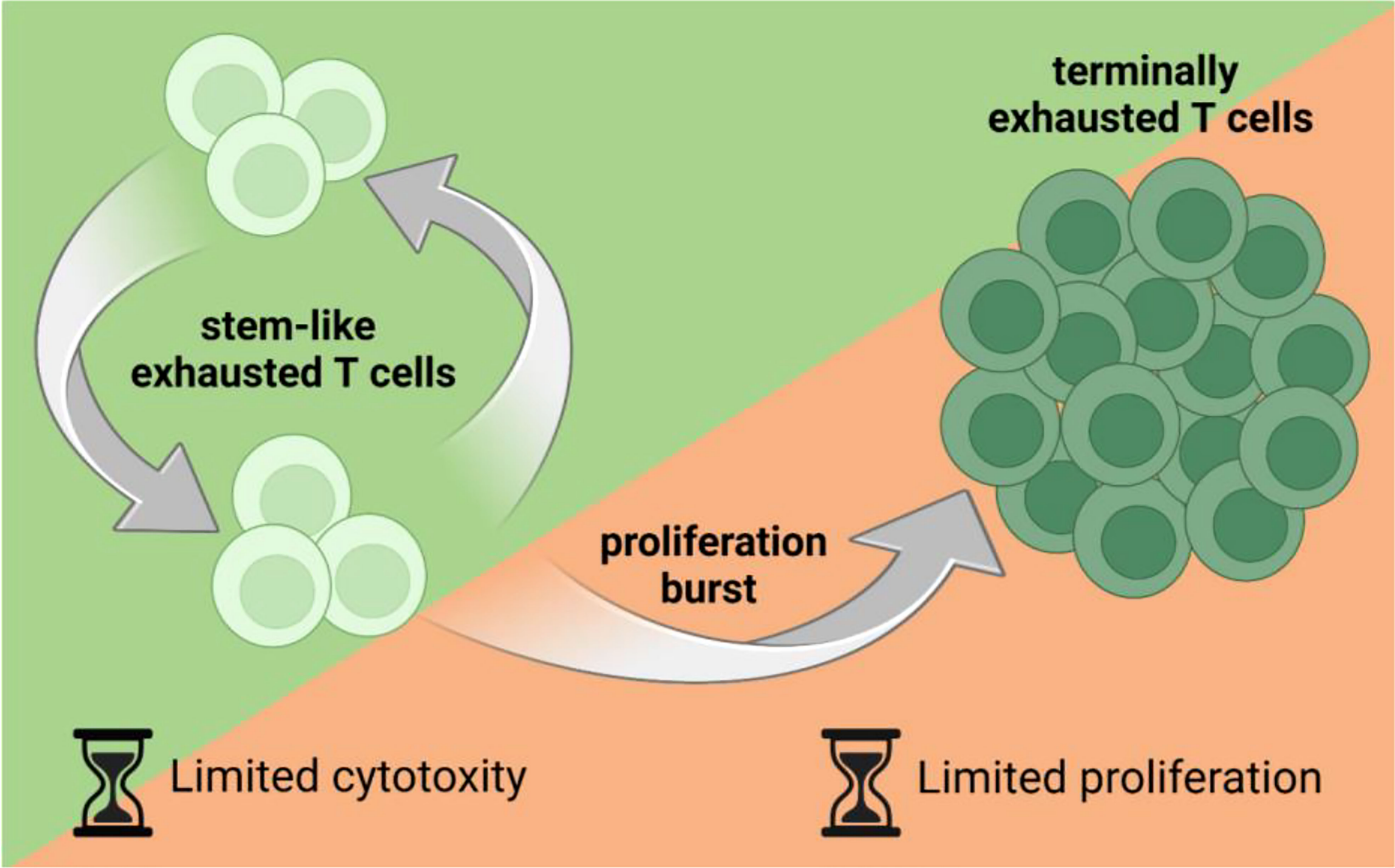
Stem-like, proliferative exhausted T cells produce progeny populations of exhausted T cells, including terminally exhausted T cells. Stem-like exhausted T cells have the capacity to proliferate but have been considered less cytotoxic than terminally exhausted T cells. On the other hand, terminally exhausted T cells have been considered more cytotoxic than stem-like exhausted T cells but have a very limited proliferation capacity. Figure created with BioRender.com.

T cell exhaustion is an umbrella term describing various functional states of T cells. Exhausted T cells can be divided into subpopulations based on functionality and phenotype, ranging from stem-like exhausted T cells to terminally exhausted T cells (7, 9, 10). However, there are no universal, clearly defined lines between the exhaustion subpopulations, and hence T cell exhaustion resembles a continuum; T cells shifting from precursor to terminal exhaustion. Some of the main markers used for categorizing populations of exhausted T cells include programmed death receptor 1 (PD-1) and transcription factor 1 (TCF-1) (10, 11). TCF-1 is a transcription factor essential for T cell development (12), and the maintenance of TCF-1 expression in exhausted cells is limited to approximately three rounds of divisions (13), where after its expression is epigenetically silenced (10). PD-1^+^TCF-1^+^ precursor exhausted T cells have stem cell-like properties as they have the capacity to proliferate, self-renew and further produce progeny populations of exhausted T cells, including terminally exhausted less proliferative PD-1^+^TCF-1^–^T cells (**Figure 1**) (11, 14). Studies have shown that the epigenetic state of exhausted T cells is irreversible (6, 9), indicating that exhaustion of T cells is a one-way process. However, recent studies suggests that the epigenetic state of exhaustion may be modifiable with the right treatments (15, 16).

Most studies investigating T cell exhaustion focus on CD8^+^ cytotoxic T cells, while exhaustion in CD4^+^ helper T cells has received less attention. The main role of CD4^+^ T cells is to support immune responses by activating and recruiting immune cells through cytokine production (17). However, similarly to CD8^+^ T cells, CD4^+^ T cells in cancerous conditions show elevated expression of IRs related to exhaustion, such as PD-1 and TIM-3 (18). CD4^+^ T cells do not typically exert cytotoxic functions, but rather promote inflammation and tumor reactivity by cytokine mediated activation of CD8^+^ T cells (18). In advanced melanoma murine models, development of a tumor reactive CD4^+^ T cell population with cytotoxic activity have been identified (19), indicating that CD4^+^ cells can obtain cytotoxic functions. The potential cytotoxicity of CD4^+^ T cells has been highlighted by Cachot et al. (20), demonstrating a CD4^+^ tumor-specific cytotoxic T cell population in cancer patients by mining single-cell RNA-sequencing datasets. The mechanisms behind CD4^+^ T cell adaptation to cancerous conditions and a possible correlation between cytotoxic CD4^+^ T cells and T cell exhaustion remains unclear.

Likewise, exhaustion in unconventional γδ T cell lineages have been reported but is less investigated than in CD8^+^ T cells. The predominant T cell type in the body is αβ T cells (here referred to simply as T cells), and these express αβ TCRs consisting of an α- and a β subunit, while γδ T cells express TCRs consisting of a γ- and a δ subunit (21). Studies provides insights in the acquirement of an exhaustion signature by γδ T cell, which has been extensively reviewed by Chen et al. (22). Furthermore, it has been reported that γδ T cells can have immunosuppressive functions, imposing immune exhaustion of antitumor αβ T cells through PD-1/PD-L1 signaling (23).

### 1.2 The cancer-immunity cycle

To portray the interactions between oncology and immunology, the cancer-immunity cycle has been introduced (24). Cancer cells are defined by genetic and cellular alterations, enabling the immune system to generate T cell responses for recognition and elimination of cancer cells. The cancer-immunity cycle term is used to describe the various necessary steps of T cell-cancer cell interactions, starting from the release of cancer cell antigens. The first step of generating an immune response is tumor antigen release from tumor cells (24). Tumor antigens are originally self-antigens, which makes their recognition by the immune system more challenging compared to foreign antigens such as those originating from pathogens (25). However, tumor antigens can be recognized by the immune system by distinguishing mutations. The higher the mutational burden, the higher the chance of successful recognition by the immune surveillance system (26). Antigens released from the tumor site are then trafficked to the blood stream and captured by APCs, which process and present antigens to T cells (24). Next step involves priming and activation of naïve T cells in LNs. APCs present processed tumor antigens to naïve T cells *via* major histocompatibility complex (MHC) molecules. Each T cell expresses TCRs, typically consisting of a α and β chain. Stochastic V(D)J gene recombination during T cell maturation results in a broad variety of TCRs, estimated to contain 10^7^-10^8^ unique signatures (27). Different TCRs recognize different epitopes presented by MHCs on the cell surface of surrounding cells. Binding of specific TCRs to MHC-antigen complexes, leads to activation and proliferation of T cells (24, 28). Activated T cells are trafficked to the tumor site *via* the blood stream guided by chemokine signaling. Chemokines are a subcategory of cytokines providing chemical signals trafficking immune cells to specific destinations (29). Infiltration of T cells to tumors is desired, since high levels of cytotoxic T cells in the TME correlates with a positive antitumor response in cancer patients (30). *Via* TCR specificity, T cells recognize antigens earlier activated against by APCs in LNs. Once TCRs of T cells bind antigens on cancer cells, T cells can kill cancer cells by releasing perforin and granzyme B (31). Killing of cancer cells triggers release of more antigens, leading to further immune responses, and so the cancer-immunity cycle continues.

## 2 Exhausted T cells in the cancer-immunity cycle

### 2.1 Priming of tumor-specific T cell activation and exhaustion in lymph nodes

The draining lymph node is the first site of APC-T cell interaction (32). Antitumor T cell response-efficacy is dependent on the quality of antigen presentation and possible immunosuppressive conditions of the microenvironment (32–34). Tissue-derived soluble antigens in lymphatic fluid flow into LNs *via* afferent lymphatic vessels (35). Antigens are captured by LN resident and migratory dendritic cells (DCs) and transported to the LN cortex and paracortex for antigen presentation to T cells (35–37). Cross-presentation of DC internalized tumor antigens to CD8^+^ T cells is a key step for priming antitumor immunity (38).

Presence of antigens is necessary for adaptive immune responses (39), and the continuous stimulation by tumor-derived antigens can be considered critical giving the ongoing evolution and accumulation of mutations in tumor cells (40–42). However, it has been shown that one of the main factors behind T cell exhaustion is prolonged, consistent antigen stimulation (43), indicating that the factors making robust immune responses against tumor cells possible are also the ones assumed to be dampening immune responses in form of T cell exhaustion. Antigenic peptides with a low mutational burden deliver weaker TCR signals (26), and are thus less potent to induce an immune response but might also be less potent drivers of T cell exhaustion. Weak TCR signaling combined with lack of co-stimulation rather leads to T cell anergy than exhaustion (44, 45), an extended hyporesponsive state considered a tolerance mechanism (46). Dysfunction in form of anergy is induced rapidly after antigen stimulation, while exhaustion is developed progressively over a period of weeks to months (47).

Studies provide evidence of the maintenance of a stem-like TCF-1^+^ CD8^+^ exhausted T cell reservoir in tumor-draining lymph nodes (TDLNs), and these stem-like exhausted T cells are necessary for long-term T cell responses and efficacy of immunotherapy (48). Data suggests that T cells differentiate towards a stem-like exhausted state in LNs, becoming prepared for migration towards tumor sites (48). Studies propose that activated CD8^+^ T cells in human TDLNs are precursors to tumor-resident stem-like CD8^+^ T cells (48). Prokhnevska et al. (49) used murine tumor models to reveal that tumor-specific CD8^+^ T cells in TDLNs were activated, but lacked an effector phenotype. When the tumor-specific stem-like CD8^+^ T cells migrated into the tumor, effector differentiation was driven by additional co-stimulation by APCs in the TME. This tumor-specific CD8^+^ T cell activation model proposes a two-step activation: initial activation in TDLNs and additional co-stimulation in the tumor, resulting in subsequent effector program acquisition (49).

Stem-like CD8^+^ T cells are also referred to as progenitor exhausted T cells (T^PEX^), and characterized to be TCF-1^+^, CXCR5^+^, PD-1^int^ and TIM-3^-^ (9). Lugli et al. (50) describes T^PEX^ cells as partially memory, effector, and exhausted T cells. T^PEX^ cells retain memory-like gene expression and preferentially localize in lymphoid tissues, but also display traits ascribed to effector-like T cells. Upon antigen stimulation, TCF-1^+^ T cells have shown contradictory IFN-γ production capacity. Some studies display increased IFN-γ production by TCF-1^+^ T^PEX^ cells compared to TCF-1^-^ terminally exhausted T cells (51), while other studies have shown greater production of IFN-γ by terminally exhausted T cells than T^PEX^ cells after *in vitro* stimulation (9).

T cell exhaustion has been well characterized in chronic infections in lymphocytic choriomeningitis virus (LCMV) models (52). In a LCMV-model study by Im et al. (53), a distinct CXCR5^+^ LCMV glycoprotein 33–41 epitope (GP33)-specific CD8^+^ T cell population was found in the spleens of mice chronically infected with the LCMV clone 13 strain. In contrast, in mice infected with the LCMV Armstrong strain having cleared the infection, GP33-specific memory CD8^+^ T cells did not express CXCR5. The study showed that during early phase chronic infection, both CXCR5^+^ and CXCR5^-^ CD8^+^ T were found in the blood, but at later stages on infection (day 30 onwards), only the CXCR5^−^CD8^+^ T cells were found in the blood (53). In the same study, the CXCR5^+^Tim-3^−^ subset displayed to be TCF-1^+^ whereas the CXCR5^−^TIM-3^+^ cells were TCF1^−^. Lymphoid tissue resident T cells were characterized by TCF-1 expression, while mainly TCF-1-negative cells were found in the periphery (53).

Dammeijer et al. (54) used LNs of ovalbumin (OVA)-expressing AE17 mesothelioma tumor mouse models to analyze the frequencies and phenotype of tumor antigen-specific CD8^+^ T cells. The study revealed that TDLN-localized tumor-specific PD-1^+^CD8^+^ T cells are capable of effectively initiate antitumor immune responses following TDLN-targeted PD-L1-blockade. Following PD-L1-blockade, TDLN-resident T cells induced T^PEX^ cell accumulation at the tumor site, resulting in improved tumor control. These findings support the pivotal role of progenitor exhausted T cells in LNs as an extra-tumoral source of antitumor T cell activity.

**Figure 2:**
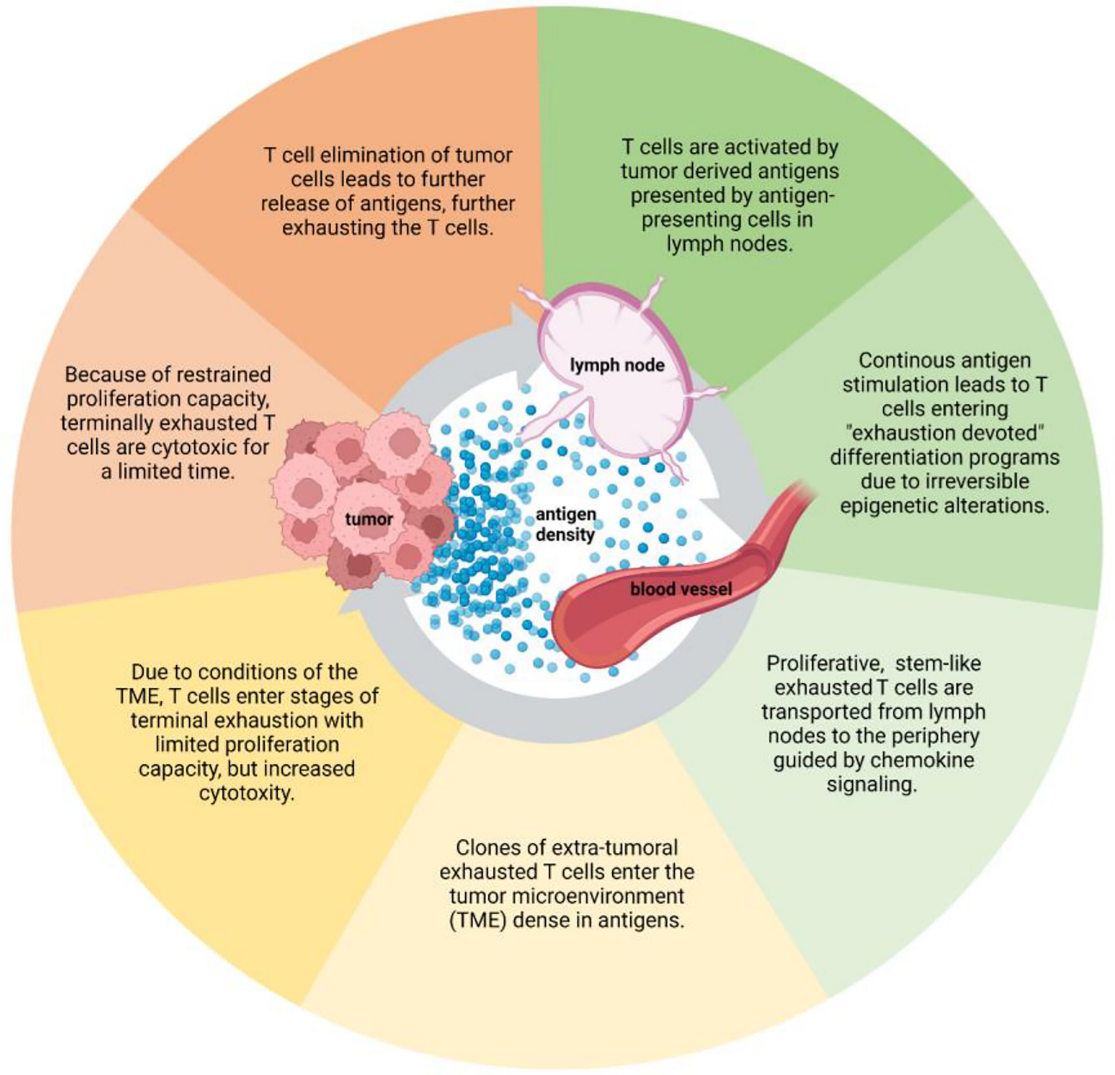
The development of T cell exhaustion throughout the cancer-immunity cycle. The exhaustion journey of T cells starts from tumor antigen-T cell interaction in lymph nodes. The exhaustion continues as the cancer-immunity cycles goes on, impacting the tumor cell elimination capacity of T cells. Figure created with BioRender.com.

These data indicate that the exhaustion journey of T cells is initiated already in the lymph nodes, preparing for later terminal stages of exhaustion (**Figure 2**). The antigen density in lymph nodes might be enough to initiate exhaustion of T cells, but not enough to promote terminal differentiation of exhausted T cells. Hence, TCF-1^+^CD8^+^ T^PEX^ cells migrating from lymph nodes to tumor sites provide a therapeutic window for maintaining a proliferative T cell population and possibly preventing differentiation of a terminal exhausted state of T cells (48, 55).

### 2.2 Migration of exhaustion committed T cells from lymph nodes to tumor sites

Activated T cells are trafficked to tumor sites *via* the blood stream guided by chemokine signaling (**Figure 2**) (29). Chemokines are secreted by various cell types in the TME, including tumor cells and immune cells. Loss of CCR7, a lymph node homing chemokine receptor, allows effector T cells to migrate to non-lymphoid tissues (33), and chemokines associated with tumor homing of T cells attract T cells to the TME. CXCL9, CXCL10 and CCL5 are positively associated with immune cell infiltration to tumors, and likewise CX3CL1, CCL3, CCL4, CCL11, and CXCL11 have been associated with T cell infiltration (55). Most chemokine profiles described above are inducible by IFN-γ, which is one of the key proinflammatory molecules secreted by effector T cells. Presence of activated T cells secreting IFN-γ in the TME cause a positive feedback loop, since IFN-γ leads to production of various chemokines attracting T cells to tumors (55). T cells reaching terminal exhaustion and thus produce less IFN-γ (2, 51), could possibly interfere with the positive feedback loop and reduce T cell attraction to the TME.

T cell trafficking is facilitated by dynamic associations between T cells, endothelial cells, and adhesion molecules (33). Lymphocyte function-associated antigen-1 (LFA-1) is an adhesion molecule expressed by activated T cells (56). Binding of LFA-1 to its ligand ICAM-1 expressed by endothelial cells, APCs and tumor cells, facilitates endothelium adhesion, prolonged contact with APCs, and tumor cell binding (57). Hence, LFA-1-ICAM-1 interactions can impact the cancer-immunity cycle from beginning to end. In lymph nodes, ICAM-1 expression on APCs is essential to guide T cell migration throughout the lymph node (58), and LFA-1-ICAM-1 engagement is necessary for complete T cell activation and differentiation (59). Studies have investigated the effect of PD-1/PD-L1 immune checkpoint blockade (ICB) on adhesion molecule expression and T cell motility. In chronic infection LCMV models, exhausted T cells had reduced motility capacity, and PD-1 blockade was observed to restore T cell motility leading to increased clearance of viruses (60). Whether the same applies for PD-1/PD-L1 blockade in tumor models remains to be investigated.

Several studies provide evidence that anti-PD-1 ICB treatment drives peripheral T cell expansion and recruitment of *de novo* responses. A study by Nagasaki et al. (61) underlines that clonotypes in exhausted tumor infiltrating lymphocyte (TIL) populations rarely overlap with non-exhausted peripheral blood leukocytes (PBLs), indicating that TDLNs might be the primary source of newly infiltrating exhausted T cells in clusters promoted by PD-1 blockade. Similar conclusions were made by Yost et al. (62), providing evidence that both pre-existing and new CD8^+^ T cell clones infiltrate tumors following PD-1 blockade. Beltra et al. (10) have identified two distinct circulating states of exhausted T cells: TCF1^+^CD69^-^ Tex^prog2^ and TCF1^-^CD69^-^T-bet^hi^ Tex^int^. The Tex^int^ population expanded upon PD-1 pathway blockade, but ultimately differentiated into a TCF1^-^CD69^+^Eomes^hi^ terminally exhausted subset. Upon PD-L1 blockade, TCF1^+^CD69^+^ Tex^prog1^ and TCF1^-^CD69^+^ Tex^term^ cells increased 2.1- and 2.2-fold, while Tex^prog2^ and Tex^int^ accumulated heavily and increased 17- and 10-fold. Results of these studies call attention to circulating exhausted T cells as the main source of expanding T cells upon ICB treatment.

Studies have described a shift from precursor to terminally exhausted T cell as T cells migrate from the lymphoid tissues to the tumor site (10). During this shift a change in transcriptional programs inducing expression alterations of transcription factors BATF, IRF4, NR4A, EOMES, NFATC1 and TOX have been identified (63–65). Especially TOX have been reported as a critical TF for exhaustion development and differentiation. TOX expression correlates with an exhausted transcriptional program of T cells, including co-expression of PD-1, TIM-3, and CD244 (66, 67). Interestingly, deletion of TOX lead to chromatin inaccessible gene regions coding for the IRs *Pdcd1, Entpd1, Havcr2, Cd244* and *Tigit* and thus reduced IR expression. Even though TOX-deleted T cells showed reduced IR expression, they have been observed to be dysfunctional in terms of effector functions such as cytokine release (66).

### 2.3 Infiltration of exhausted T cells to the TME and functions of T cells in the TME

#### 2.3.1 T cell tumor infiltration and cytotoxicity

Studies provide evidence of pre-existing TILs having limited reinvigoration potential following ICB treatment, and the majority of ICB responsive cells originating from outside tumors (62). Profiling of T cells in human basal and squamous cell carcinomas prior and post anti-PD-1 treatment displayed a more robust clonal expansion of CD8^+^ T cell with an exhausted phenotype compared to other TIL populations (62). Additionally, clonal expansion of T cells in response to immunotherapy was derived from extra-tumoral clones of T cells, and the effect was specific to T cells characterized as exhausted (62). This study demonstrates a tumor infiltration capacity of particularly exhausted T cells. However, where on the exhaustion continuum such capacity is at its most optimal remains to be investigated.

T^PEX^ CD8^+^ TILs are able to control tumor growth and can respond to anti-PD-1 therapy, while terminally exhausted TILs cannot (9). As pre-existing TILs characterized as terminally exhausted have been considered resistant to ICB immunotherapy (9, 62, 68), recent studies have investigated whether these terminally exhausted T cells are involved in generation of an immunosuppressive TME (68). Vignali et al. (68) have identified intratumoral CD8^+^ terminally exhausted T cells with transcriptional features of CD4^+^Foxp3^+^ regulatory T (Treg) cells and show that these cells are capable of directly suppressing T cell proliferation ex vivo.

Eradication of tumor cells can be considered the final goal of the cancer-immunity cycle. Cytotoxic mechanisms of T cells rely on two distinct pathways: the perforin-granzyme-induced apoptosis pathway (granule exocytosis) and Fas/Fas ligand (FasL) pathway (death ligands) (69, 70). Out of these two pathways, granule exocytosis is considered the main pathway to eliminate cancer cells (69, 70). Perforin is a pore-forming protein facilitating the delivery of granzymes into target cells (71), and granzymes belong to a family of serine proteases known for mediating cytotoxic T cell elimination of infected cells and tumor cells (72). A study by Wu et al. (73) demonstrated defects in CD8^+^ TIL perforin expression in colorectal cancer (CRC), even though these cells stored the highest levels of granzyme B. Furthermore, PD-1 expression correlated with impaired perforin production, and the intact perforin expression was restricted to tumor resident T cells (73). Yan et al. (74) have identified an effector memory phenotype PD-1^+^CXCR1^+^ CD8^+^ T cells population able to withstand chemotherapy and expand after chemo immunotherapy with cytolytic activity, defined by granzyme B and perforin release. Hurkmans et al. (75) studied granzyme B serum levels in metastatic non-small cell lung cancer (NSCLC) patients post PD-1 blockade by Nivolumab. Lower serum levels of granzyme B correlated with worse clinical outcome, and interestingly, serum levels of granzyme B positively correlated with peripheral abundance of T cell populations expressing PD-1 and TIM-3. Together these findings show sustained and elevated granzyme B release capacity of exhausted T cells, while perforin production remains questionable. It is widely assumed that both perforin and granzyme B are necessary for efficient cytotoxicity of T cells. Interestingly, perforin knockout OT-I T cells have been shown to efficiently kill MC38^Ova^ and B16^Ova^ cells in prolonged presence (>18 hours) of antigens (76), indicating cytolytic activity of T cells in absence of perforin.

The Fas/FasL pathway plays an essential role in regulating apoptosis and T cell activation (77). The Fas/FasL signaling in exhausted antigen-specific CD8^+^ T cells during tumor immune response have been studied in C57BL/6 mice inoculated with EG.7. The study showed that the number of activated antigen-specific CD8^+^ T cells decreased *via* apoptosis during prolonged tumor immune responses, but the number of T cells in FasL-dysfunctional gld mice were higher than in control mice (78). Thus, the Fas/FasL signaling pathway is critical for survival of exhausted CD8^+^ T cells during tumor immune response. In chronic infection LCMV mice models, the FasL mRNA expression is reported to be substantially elevated in exhausted CD8^+^ T cells compared to naive, effector, and memory CD8+ T cells, and the Fas/FasL pathway is discussed to provide alternate cytolytic mechanisms during chronic infection (1). In CD8^+^ T cells specific for chronic persistent virus (HIV), PD-1 expression has been observed to be associated with spontaneous and Fas-induced apoptosis (79). Studies provide insights of an altered Fas/FasL pathway in exhausted T cells, but the precise outcomes of these alterations in cancerous conditions remain to be investigated.

Studies describe various ICAM-expression levels by exhausted T cells, and its impact on tumor eradication remains unclear. LFA-1-ICAM-1 binding enables T cell-tumor cell binding prior to contact killing by cytotoxic elimination. The importance of proper immune synapse formation prior to target cell elimination has been highlighted in cancer models of hematological malignancies. Ramsay et al. (80) show that F-actin polymerization was suppressed upon T cell chronic lymphocytic leukemia (CLL)-B cell encounter, leading to impaired immune synapse formation, and hindered antitumor activity. Studies show that loss of ICAM-1 expression as a possible consequence of reduced proinflammatory cytokine production (81, 82), could lead to elevated granzyme B release (83). Loss of ICAM-1 expression has been observed to result in elevated levels of IFN-γ and granzyme B, as well as enhanced cytotoxicity (83). T cell activation without classical immune synapse formation but *via* TCR microclusters has been reported (84, 85), providing evidence of maintained T cell activation and effector functions regardless expression levels of LFA-1 and ICAM-1.

#### 2.3.2 T cell metabolic activity

To adapt to hypoxic conditions in the TME, T cells undergo a metabolic switch upon activation. Metabolic reprogramming is essential to meet the increasing energy demand necessary for effector T cell functions (86). Naïve T cells exhibit lower energy demands than effector T cells and rely on oxidative phosphorylation (OXPHOS) derived ATP as the main source of energy (87). Effector T cells utilize aerobic glycolysis as their primary metabolic program, increasing the glucose uptake and oxidative consumption (88). T cells can also use fatty acids as an energy source, and the use of fatty acid β-oxidation (FAO) pathway is linked to memory T cell differentiation (89). Furthermore, FAO is an important pathway of survival in metabolically stressed cells (89), and Tregs generally rely upon FAO for their metabolic needs (90). The metabolic plasticity and regulation of T cell exhaustion has been extensively reviewed by Li et al. (91), focusing on T cell exhaustion in chronic infections. FAO and OXPHOS are discussed to be the primary energy sources for T^PEX^, while terminally exhausted T cells rely on glycolytic metabolism, but having reduced glucose uptake and an inability to effectively utilize OXPHOS to provide energy (91).

The high consumption of glucose by cancer cells creates glucose derivatized, acidic extracellular conditions in the TME (92), caused by lactate accumulation (93) known to suppress the proliferation and effector functions of cytotoxic T cells (94). Effector T cells exhibit decreased mitochondrial respiratory activity, and studies show exhausted T cells exhibiting suppressed glycolysis and mitochondrial respiration, causing poor metabolic fitness and exhaustion (95, 96). A study by Chang et al. (88) displays the role of aerobic glycolysis as a metabolically regulated signaling mechanism, and activated T cells blocked from aerobic glycolysis have a compromised ability to produce IFN-γ (88), possible explaining decreased IFN-γ production of terminally exhausted T cells in the TME. A study by Scharping et al. (97) point out that progressive loss of mitochondrial function and mass correlates with decreased cytokine production in solid tumor infiltrating T cells. Expression of the glucose transporter-1 (Glut1) has been observed to increase in PD-1^hi^ exhausted T cells in hepatitis B virus (HBV) chronic infection, and these cells were dependent on glucose supplies. In contrast, non-chronic infection cytomegalovirus (CMV)-specific T cells that could utilize OXPHOS in the absence of glucose to optimize their energy supply (98). Similar metabolic deficiencies in exhausted T cells have not yet been investigated in tumor models.

The metabolism of T cells has been studied in murine melanoma models, and a positive correlation between immune checkpoints expression levels on CD8^+^ T cells and total cholesterol content in the cells has been observed. Lung B16 tumor-infiltrating PD-1^high^2B4^high^ CD8^+^ T cells had significantly higher cholesterol content than PD-1^med/low^2B4 ^med/low^CD8^+^ T cells. The same pattern was observed in LN and spleen resident T cells, although these cells had significantly lower cholesterol content than the tumor-infiltrating T cells (99). Cholesterol is a tumor metabolic byproduct inducing metabolic stress in T cells, and the study demonstrated that cholesterol in the TME induces CD8^+^ T cell exhaustion *via* activation of the endoplasmic reticulum (ER)-stress sensor XBP1. XBP1 can directly increase PD-1, TIM-3, and LAG-3 expression, thus immunosuppression of T cells (99). Patsoukis et al. (100) discovered the incapability of activated T cells to engage in glycolysis upon PD-1 ligation, and their studies show that PD-1 ligation increase the FAO rate. FAO supports T cell persistence, but not necessarily T cell function. Altogether these findings support the theory of an altered metabolic program in exhausted T cells, causing altered effector functions, but precise locations and timelines of metabolic switches in exhausted T cells during the cancer-immunity cycle remain to be explored.

## 3 T cell exhaustion as a functional adaptation to persistent antigen stimulation

T cell exhaustion has been discussed to be an evolutionarily conserved adaptation to chronic antigen stimulation, important for restricting autoreactivity and immunopathology. RNA-sequencing and ATAC-sequencing data generated from circulating T cell subsets from healthy donors show that circulating PD1^+^ CD39^+^ T cells had the highest enrichment of tumor exhausted T cell genes, providing transcriptional evidence of recent proliferation and characteristics of intermediate exhausted T cells (101). This study provides evidence of circulating exhausted T cell populations present also in healthy donors, suggesting that PD-1^+^CD39^+^CD8^+^ exhausted T cells are not restricted to cancer or chronic infections (101). Their existence might point towards an alternative fate to deletion for autoreactive T cells, hence enabling a broader diversity of T cell populations, but their precise role in healthy donors remains to be explored. Galletti et al. (102) have identified two different subsets of human stem-like CD8^+^ memory T cell progenitors in healthy donors: CCR7^+^PD-1^−^TIGIT^−^ stem-like T (T^STEM^) cells and CCR7^+^ PD-1^+^TIGIT^+^ T progenitor exhausted T (T^PEX^) cells. The T^PEX^ population gave rise to a cell linage with reduced functionality compared to T^STEM^ cells. Interestingly, the T^PEX^ cells had memory-like features, suggesting that exhaustion-like and memory-like characteristics could coexist in healthy individuals. This further indicates that T cell exhaustion could be a functional adaption to persistent antigen stimulation aimed at minimizing the risk of immunopathology, and simultaneously maintain a memory-like population. The role of exhaustion in limiting immunopathology is underlined in a study by McKinney et al. (103), displaying reduced clinical autoimmunity as T cell exhaustion gets more severe.

Expression of members of the transcription factor (TF) family NR4A is regulated downstream of TCR signaling (104). Beltra et al. (10) have defined a four-cell-stage developmental framework for exhausted T cells. In studies combining motif enrichment analysis with RNA expression, NR4A2 was predicted to be the most enriched TF in terminally exhausted T cells. TCR stimulation induces expression of the NR4A family (105), indicating that terminally differentiated exhausted T cells are subjects of heavy TCR signaling. *Nr4a* triple knockout (TKO) in CAR T cells have been shown to prolong the survival of tumor-bearing mice, and NR4A inhibition has been pointed out as a promising cancer immunotherapy strategy (14). Expression of mRNAs encoding effector proteins such as granzymes, TNF and IL-2Rα were increased in *Nr4a* TKO TILs (14), indicating that dense antigen environments and heavy TCR signaling eventually leads to decreased cytotoxicity of T cells. Together these findings point out that prolonged antigen-TCR stimulation indeed leads to a terminally exhausted state of T cells and eventual loss of cytotoxicity, limiting the risk of immunopathology but also limiting tumor cell clearance. These studies draw attention to the importance of eliminating tumor cells within an exhaustion restricted time frame.

Studies suggest that varying tumor types might generate distinct exhaustion patterns in T cells. Woroniecka et al. (106) performed a study with mouse-implanted SMA-560 malignant glioma, CT2A malignant glioma, E0771 breast medullary adenocarcinoma, B16F10 melanoma, and Lewis Lung Carcinoma (LLC), comparing exhaustion patterns according to cell line. They found distinct functional programs of exhausted T cells depending on tumor type, displaying variations in proinflammatory cytokine production and expression of IRs. Tumors causing greater functional impairment of T cells showed higher levels of alternative IRs such as TIGIT, CD39 and 2B4, indicating that greater functional impairment indeed provides opportunities for alternative immunotherapy strategies. Distinct exhaustion programs according to tumor type explains varying response to PD-1 pathway inhibitors, which have shown greatest results in melanoma, NSCLC, RCC, and metastatic bladder cancer (107). These concluding remarks point out the need to understand not only how T cell exhaustion impacts the cancer-immunity cycle, but the varying T cell exhaustion cycles according to specific tumor types. Distinct exhaustion programs according to tumor type calls attention to the dynamic features of T cell exhaustion, adapting to specific tumor conditions.

As the cancer-immunity cycle points out, the balance between stimulating factors and inhibitors is critical (24). Tumor cells can exploit immune escape mechanisms such as loss of antigenicity, loss of immunogenicity or generation of immunosuppressive microenvironments, to avoid elimination by the immune system (108). In a similar manner, T cell exhaustion could be regarded as an alternative mechanism of the immune system, a tool to control cancer. Exhausted T cells aren’t suitable tools for all types of cancer types and conditions, hence having a contrary role, in some circumstances acting as an asset and in some as a liability.

## 4 Potential immunotherapeutic interventions for reinvigoration of exhausted T cells

Immune checkpoint blockade (ICB) therapies such as antibodies targeting PD-1, PD-L1, CTLA-4 and LAG-3 aim to activate T cells by interrupting inhibitory signals (**Table 1**). Today, the focus area of controlling T cell exhaustion lies within preventing exhaustion and reactivating already exhausted T cells (131). Combination with traditional ICBs PD-1, PD-L1 and CTLA-4 and second-generation checkpoint targets such as LAG-3, TIM-3 and TIGIT are being investigated (131). Since the first ICB therapy was approved in 2011, the US Food and Drug Administration (FDA) have issued over 65 approvals for 20 types of neoplasms (132). Studies from the past decade provide evidence of checkpoint therapies being most efficient when administering in an early state of exhaustion (107). Despite increased knowledge in targeting T cell exhaustion and the large number of FDA approvals, only 20.2% of patients receiving ICBs achieve objective response, and out of these only 13% of patients achieve durable responses for multiple years (132). To improve patient responses, combination therapies with ICBs can be utilized. FDA approved therapies to be combined with ICBs include chemotherapy, radiation, anti-angiogenic agents, cancer vaccines and adoptive cell therapies (ACT) (133).

**Table 1:** Current and potential future immunotherapeutic T cell exhaustion targets.

Symbol	Target	Biological context	Status	References
PD-1	Programmed cell death protein 1	Negative regulator of immune cell activity.	FDA approved	(109, 110)
PD-L1	Programmed cell death protein ligand 1	Negative regulator of immune cell activity.	FDA approved	(109, 110)
CTLA-4	Cytotoxic T-lymphocyte associated protein 4	Negative regulator of immune cell activity.	FDA approved	(110, 111)
LAG-3	Lymphocyte activation gene 3	Negative regulator of immune cell activity.	FDA approved	(110, 112)
TIGIT	T cell immunoreceptor with immunoglobulin and ITIM domain	Negative regulator of immune cell activity.	Clinical trials	(113, 114)
TIM-3	T cell immunoglobulin mucin 3	Negative regulator of immune cell activity.	Clinical trials	(115)
BTLA	B and T lymphocyte attenuator	Negative regulator of immune cell activity.	Clinical trials	(116, 117)
VISTA	V-type immunoglobulin domain-containing suppressor of T cell activation	Negative regulator of immune cell activity.	Clinical trials	(117, 118)
ICOS/CD278	Inducible T-cell co-stimulator	Co-stimulatory regulator of immune cell activity.	Clinical trials	(117, 119)
GITR	Glucocorticoid-induced tumor necrosis factor receptor–related protein	Co-stimulatory regulator of immune cell activity.	Clinical trials	(120)
4-1BB/CD137/TNFRSF/ILA	Tumor necrosis factor receptor superfamily member 9	Co-stimulatory regulator of immune cell activity.	Clinical trials	(121)
OX40/CD134	Tumor necrosis factor receptor superfamily member 4	Co-stimulatory regulator of immune cell activity.	Clinical trials	(122)
NFAT	Nuclear factor of activated T cells	Transcription factor. Regulates effector functions and exhaustion of T cells.	Early discovery	(123)
BATF	Basic leucine zipper ATF- like transcription factor	Transcription factor. Regulates effector functions and exhaustion of T cells.	Early discovery	(123)
IRF4	Interferon regulatory factor 4	Transcription factor. Regulates effector functions and exhaustion of T cells.	Early discovery	(123, 124)
TOX	Thymocyte selection-associated high-mobility group box	Transcription factor. Regulates effector functions and exhaustion of T cells.	Early discovery	(67, 125)
NR4A	Nuclear receptor subfamily 4 group A	Transcription factor. Regulates effector functions of T cells.	Early discovery	(14)
c-JUN	Transcription factor Jun	Transcription factor. Regulates effector functions of T cells.	Early discovery	(126)
PTPN2	Protein tyrosine phosphatase non-receptor type 2	Phosphatase. Regulates cell development.	Early discovery	(127)
CD39	Ectonucleoside triphosphate diphosphohydrolase-1	Adenosine receptor. Upregulated in response to various stress stimuli.	Early discovery	(128)
Vps4b	Vacuolar protein sorting- associated protein 4B	ATPase. Participates in vesicular trafficking and autophagosome maturation.	Early discovery	(129)
1α,25(OH)2D3	1α,25(OH)2D3	Active form of vitamin D. regulates expression of Pdcd1, Tim3, and Tigit genes.	Early discovery	(130)

Some tumors have been shown particularly challenging to target due to T cell exhaustion. Chimeric antigen receptor (CAR) T cells have been proven to be a potent tool for targeting blood cancer, but the dense antigen environment in solid tumor often generates severe exhaustion of these cells, causing restricted efficacy. Studies have shown that combination therapies with ICBs and CAR T cells can improve T cell expansion and tumor reactivity (134).

To further improve CAR T cell efficacy, studies have targeted the exhaustion inducing transcription factor protein TOX (135). TOX is upregulated and co-expressed with PD-1, TIM-3, and CD244 in non-Hodgkin lymphoma (136), multiple myeloma (137), and in acute myeloid leukemia patients (138). Depletion of the *Tox* gene in CAR T cells proved to improve persistence and tumor reactivity in tumor inoculated mice (139). Recent studies point out the transcription factor MYB as an orchestrator of T cell exhaustion. MYB mediates differentiation of a CD62L^+^ T^PEX^ cell stem-like population. Upon PD-1 ICB treatment this population is responsible for the proliferative burst, and thus, MYB could play a central role in therapeutic checkpoint blockade success (140). Other transcriptions factors found to promote exhaustion are NFAT and NR4A, while BATF attenuates exhaustion (141). The transcription factor IKZF3 is a repressor of IL-2 (142), and Hay et al. (2022) used an *in vitro* model to show that exhausted T cells partially can be rescued by treatment with Lenalidomide, an IKZF3 small molecule inhibitor, either as a single treatment or in combination with ICB treatment.

As exhausted T cells display dysregulation in mitochondrial function, strategies for improving mitochondrial biogenesis have raised an interest. In B16^OVA^ tumor microenvironments, PGC1α overexpressing OT-I T cells, leading to increased mitochondrial mass and OXPHOS, are enriched (97). These cells displayed enhanced tumor efficacy, demonstrating a preservation of T cell function in the TME by reprogramming these cells to favor mitochondrial biogenesis. Metabolic stress, such as hypoxia and glucose deprivation in the TME of solid tumors, have shown to lower tumor immunogenicity (143). To improve responses to ICB, signaling pathways such as the PI3K/AKT pathway, could be targeted to restore tumor cell recognition by T cells (143). Thus, addressing metabolic and signaling pathways hold promising potential to improve immunotherapy responses.

The posttranscriptional mechanisms regulating exhaustion of T cells remains poorly understood. Using Affymetrix miR arrays, the microRNA (miR) expression in isolated LCMV DbGP33–41–specific CD8^+^ T cells have been examined (144). MiR-29a was identified as an exhaustion attenuating molecule in chronic infection and could also play a role in decreasing exhaustion in cancer. Data suggests that miR-29a attenuates TCR signaling pathways involved in driving exhaustion and thus, enhanced expression of miR-29a favor a durable T memory cell-like rather than exhausted T cell-like differentiation in conditions of persistent antigen stimulation (144). Hence, targeting microRNAs in combination therapies could represent novel solutions for improved immunotherapy efficacy.

The development and maintenance of exhausted T cells can be controlled by manipulation of molecular pathways, by utilizing methods such as CRISPR gene editing. Using CRISPR, synthetic DNA sequences can be inserted to generate next-generation autologous T cell therapies (145), and there is an interest in modifying T cell exhaustion promotors such as *Tox* for augmented tumor control (131). Other proposed CRISPR targets attracting attention by biotech and pharmaceutical companies are PTPN2, NR4A and c-JUN (126, 127, 131). Unlike most molecules and genes of interested currently being targeted to prevent T cell exhaustion, deletion of *Ptpn2* has shown to scale up the population of terminally exhausted T cells whilst preserving the population of self-renewable progenitor exhausted T cells (127).

To increase and prolong patient response and survival, bispecific antibodies (BsAbs) have been introduced to the field of immunotherapy. BsAbs can bind two distinct targets on the same cell at the same time, enabling additive effects and novel therapeutic approaches (146). Dual BsAbs targets include immune checkpoints, signaling pathways, tumor associated antigens and cytokines (147). OSE Immunotherapeutics have developed a bispecific antibody checkpoint inhibitor BiCKI^®^ platform, with the BiCKI^®^-IL-7 program targeting PD-1 and simultaneously releasing IL-7, promoting TCF-1^+^ stem-like T cell expansion (148). In September 2022, Roche announced taking over Good Therapeutics’ PD1-regulated IL-2 receptor agonist program (149). Similarly to IL-7, IL-2 have potential immunostimulating and antineoplastic functions, but unregulated IL-2 release can cause toxicity. Linkage of anti-PD-1 to an alternative form of IL-2 (IL-2v) enables precise IL-2 stimulation, thus minimizing side effects and toxicity (150).

## 5 Discussion and conclusions

Throughout the cancer-immunity cycle, T cells are subjects of a variety of factors such as prolonged antigen stimulation and altered conditions of the TME, unavoidably shifting the T cells towards an exhausted state (7). Studies discuss the possibility of separate differentiation programs of T cells, one initiating the “classical” effector/memory T cells pathway and the other one initiating an exhaustion program of T cells (102). Insights display the close integration of the cancer-immunity cycle and T cell exhaustion, pointing out the need of a deeper understanding of the T cell exhaustion cycle to enable discovery of novel immunotherapies.

T cell exhaustion has potential benefits, but also consequences. A stem-like TCF-1^+^ exhausted T cell state maintain a memory-like population with proliferation capacity (10, 11). Stem-like TCF-1^+^ T cells giving rise to terminally exhausted cytotoxic T cells could serve as a type of safety mechanism. Excessive antigen density in the TME leading to short-term cytotoxic states restrain cytotoxicity to tumors sites and thus limits immunopathology in healthy tissues. Since terminally exhausted T cells have reduced proliferation capacity (10, 11), the damage caused by this population is limited. On the other hand, high cytotoxicity is desired for tumor elimination, and thus short-term cytotoxicity and lacking proliferation capacity simultaneously serves as a liability. Restricted rounds of proliferation by stem-like exhausted T cells and restricted periods of cytotoxicity creates a time frame of tumor cell clearance. If events in the exhaustion cycle exceed these time frames, the consequences of T cell exhaustion could result in tumor immune escape, rather than tumor elimination. To fully utilize the potential assets of exhausted T cells in terms of immunotherapies, the master mechanisms and regulators behind T cell exhaustion needs to be further elucidated. Nevertheless, it might be useful considering T cell exhaustion as a functional adaptation rather than simply a dysfunction.

## Author contributions

AB performed the literature review and wrote the manuscript. RL, AA, and AT provided critical discussion, revised, and approved the manuscript. AT conceived the review article.
